# Insulin-like growth factor 2 mRNA binding protein 3 (IGF2BP3) promotes lung tumorigenesis via attenuating p53 stability

**DOI:** 10.18632/oncotarget.21280

**Published:** 2017-09-27

**Authors:** Wei Zhao, Dan Lu, Liang Liu, Juan Cai, Yu Zhou, Ying Yang, Yu Zhang, Jun Zhang

**Affiliations:** ^1^ Department of Immunology, School of Basic Medical Sciences, Peking University Health Science Center, Key Laboratory of Medical Immunology, Ministry of Health (Peking University), Beijing, 100191, P.R. China; ^2^ Present address: Department of Clinical Laboratory, China-Japan Friendship Hospital, Beijing 100029, P.R. China; ^3^ Institute of Systems Biomedicine, School of Basic Medical Sciences, Peking University Health Science Center, Beijing 100191, P.R. China

**Keywords:** IGF2BP3, lung cancer, proliferation, USP10, p53

## Abstract

Insulin-like growth factor 2 mRNA binding protein 3 (IGF2BP3/IMP3/KOC), initially identified as an RNA-binding protein, is highly expressed in embryonic tissues and a variety of cancers. Previously, our group reported that IGF2BP3 may serve as a potential diagnostic marker for lung cancer. However, little is known about the function of IGF2BP3 in lung cancer development. Here we demonstrate that IGF2BP3 expression was markedly increased in lung cancer tissues compared to normal tissues at both mRNA and protein levels. Overexpression of IGF2BP3 in lung cancer cells promoted cell proliferation, tumor migration and invasion *in vitro* and *in vivo*, whereas knockdown of IGF2BP3 exhibited opposite effects. Notably IGF2BP3 was directly associated with a deubiquitinase Ubiquitin specific peptidase 10 (USP10) and attenuated its function in stabilizing p53 protein. Silencing IGF2BP3 expression in lung cancer cells consistently increased the half-life and protein level of p53 and induced G0/G1 arrest. Thus, our data together demonstrate that IGF2BP3 promotes lung tumorigenesis via attenuating p53 protein stability.

## INTRODUCTION

Lung cancer is the leading cause of cancer related mortality worldwide [1]. Morphologically, lung cancer has two major types: non-small cell lung cancer (NSCLC) and small cell lung cancer (SCLC). Most lung cancers (85%) are NSCLC, which can be further divided into adenocarcinoma (ADC), squamous cell carcinoma (SCC) and large cell carcinoma (LCLC) [2, 3]. Although surgical resection and chemotherapy represent the major therapeutic means for lung cancer, most lung cancer patients may be inoperable at the time of being diagnosed with advanced disease grading and metastasis at regional or distal sites. Among them, a lot of lung cancers are resistant to chemotherapy [3, 4]. Therefore, understanding the contributors and identification of novel therapeutic targets in lung tumorigenesis may facilitate the development of novel drugs for lung cancer therapy.

The mammalian insulin-like growth factor-II messenger RNA-binding protein (IGF2BP) family consists of three members, which were designated as IGF2BP1, IGF2BP2 and IGF2BP3 respectively [5]. These proteins share structure homology and functional similarity. In structure, they all contain two N-terminal RNA recognition motifs (RRM) and four C-terminal nuclear ribonucleoparticles K homology (KH) domains [6]. IGF2BP3 was first reported to bind the leader 3 of 5’ UTR of IGF2 mRNAs and promote the translation of IGF2 mRNA [7]. Subsequent studies found that IGF2BP3 functions as an oncofetal protein and is up-regulated in a variety of cancers such as breast cancer, hepatocellular carcinoma, *etc* [8–19]. Now, the validated target mRNAs of IGF2BP3 include *H19, ACTB, myc, CD164, MMP9, ABCG2, PDPN, HMGA2*, *BCRP*, *SNAI2 (SLUG)*, *EIF4E-BP2*, *cyclins D1, D3 and G1* (*CCND1, D3* and *G1*), *etc* [18, 20–25]. To our knowledge, IGF2BP3 promotes cancer cell proliferation by binding the mRNAs of *IGF2, HMGA2, CCND1, D3, G1* [24, 26, 27] and enhances the invasive potential of tumor cells by stabilizing the mRNAs of *CD164, MMP9 and PDPN* [9, 28]. Besides, IGF2BP3 was also reported to be involved in tumor initiation. It can directly promote the stem-like features in triple-negative breast cancer by modulating *SLUG* [23]. Thus, IGF2BP3 was supposed to promote tumorigenesis mainly depends on its mRNA binding capacity and positively modulating transcript levels of oncogene.

The increased protein expression of IGF2BP3 in lung cancer was also documented. Immunohistochemistry showed that IGF2BP3 was expressed in 27–55% of cases of primary pulmonary adenocarcinoma and in 75–90% of cases of squamous cell carcinoma of the lung [29]. High and strong expression of IGF2BP3 is associated with moderately/poorly differentiated lung cancer and predicts poor prognosis [30, 31]. In our previous study, autoantibodies against IGF2BP3 were observed in a few fractions of patients with IGF2BP3-positive lung cancer [32]. Data currently available by us and others suggest that IGF2BP3 in lung cancer may be of diagnostic or prognostic values. However, its function in lung cancer progression remains to be explored. In the current study, we identified an unknown function of IGF2BP3 in lung tumorigenesis, by which IGF2BP3 attenuated the protein stability of p53 independent of its mRNA binding activity.

## RESULTS

### IGF2BP3 is upregulated in lung cancer tissues and cell lines

Although IGF2BP3 is an oncofetal protein, which is frequently up-regulated in a variety of cancers, the DNA copy number change of it is not yet reported. We first analyzed IGF2BP3 DNA copy numbers in lung cancer tissues by two DNA datasets in the Oncomine database. Elevated IGF2BP3 DNA copy numbers were observed in lung adenocarcinoma, squamous cell lung carcinoma, and mixed types of lung cancer compared to normal lung tissues in TCGA lung dataset and Weiss lung dataset (Supplementary Figure 1A and 1B). Besides, elevated IGF2BP3 mRNA levels were also observed in adenocarcinoma, squamous cell carcinoma and large cell carcinoma compared to normal lung tissues in the Hou and Landi lung datasets, respectively (Supplementary Figure 1C and 1D). These data together suggest that both DNA copy of IGF2BP3 gene and IGF2BP3 mRNA were upregulated in lung cancer tissues.

**Figure 1:**
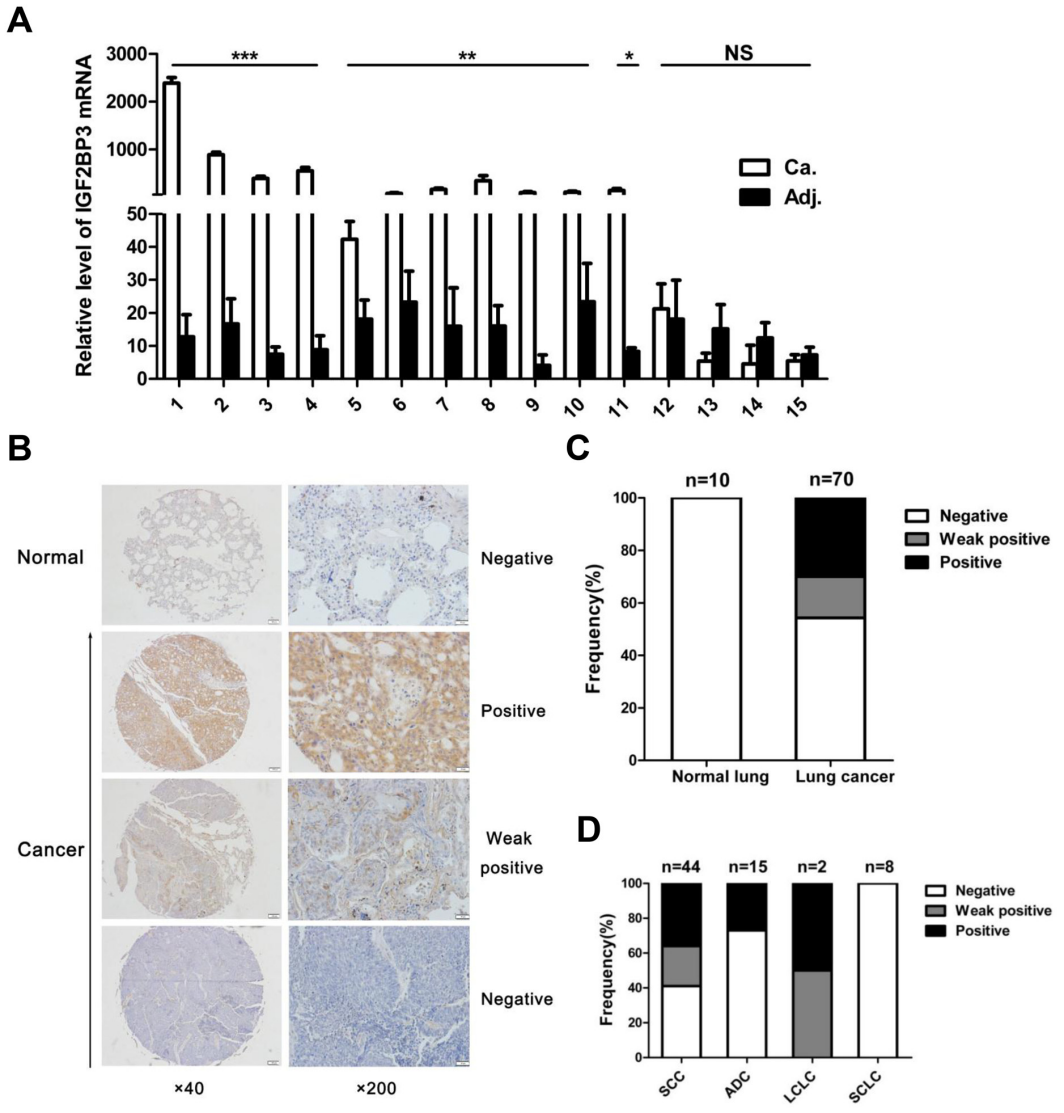
Increased expression of IGF2BP3 in lung cancer tissues (**A**) Quantitative real-time PCR for IGF2BP3 mRNA expression in 15 pairs of lung cancer versus adjacent non-cancerous tissues. IGF2BP3 expression was normalized against GAPDH. Relative levels were calculated for each sample, and a value of 1 was assigned for cancer tissue of patient 14. Experiments were repeated in triplicate. Data from one representative experiment are presented as mean ± SD. Ca, Cancer tissues; Adj, adjacent non-cancerous tissues. (**B**) Representative results of immunohistochemical staining of IGF2BP3 in normal lung versus lung cancer tissues. Magnification, ×40, ×200. (**C**) Analysis of IGF2BP3 expression in the lung cancer tissue array. (**D**) Correlation between IGF2BP3 expression and the pathological histology type of lung cancers. SCC, Squamous cell carcinoma; ADC, Adenocarcinoma; LCLC, Large cell lung cancer; SCLC, Small cell lung cancer. NS, no significance; *P < 0.05; **P < 0.01; ***P < 0.001.

To further confirm the high expression of IGF2BP3 in lung cancer tissues, we performed Real-time PCR and immunohistochemical analysis to assess the expression of IGF2BP3 in lung cancer at both mRNA and protein levels. In fifteen paired lung cancer and non-cancerous lung tissues, IGF2BP3 mRNA expression was upregulated in 6 out of 8 adenocarcinoma and 6 out of 7 squamous cell carcinoma tissues as compared to adjacent non-cancerous tissues (Figure 1A). Besides, IGF2BP3 was highly expressed in a variety of cancer cell lines including lung cancer cell lines (Supplementary Figure 2A and 2B). Among lung cancer cell lines detected, highest expression of IGF2BP3 protein was observed in A549 cells and the lowest expression was observed in H460 cells. We next examined IGF2BP3 protein expression in a tissue array containing 10 normal and 70 lung cancer tissues. No expression was detected in 10 normal lung tissues (Figure 1B). In contrast, positive expression of IGF2BP3 was observed in 32 (45.7%) lung cancer tissues (Figure 1C). Among them, positive staining was attributed to 4 out of 15 adenocarcinoma and 26 out of 44 Squamous cell carcinoma tissues (Figure 1D). Consistent with previous studies [33], positive staining of IGF2BP3 was observed both in nucleus and cytoplasm in cancer tissues (Figure 1B). The correlation of IGF2BP3 protein expression with clinical and pathologic features of the patients was summarized in Table 1. Statistical analysis indicated that the protein level of IGF2BP3 was increased in malignant lung tissues compared to normal tissues (*P* = 0.019). And there was no correlation between the protein expression of IGF2BP3 with the patients’ age and gender. Notably, IGF2BP3 is prone to be expressed in high grade of lung cancer (*P* = 0.047). Histologically, IGF2BP3 is more likely to be expressed in squamous cell carcinoma and adenocarcinoma.

**Table 1:** IGF2BP3 protein expression and clinicopathologic characteristics in lung cancer tissue microarray

Clinico-pathological characteristics	Categorization	*n*	IGF2BP3 protein expression			*P* value
			Negative	Weak positive	Positive	
Normal Lung		10	10 (100%)	0	0	0.019
Lung Cancer		70	38 (54%)	11 (16%)	21 (30%)	
Age	< 60	40	23 (58%)	6 (15%)	11 (27%)	0.82
	>= 60	30	15 (50%)	5 (17%)	10 (33%)	
Gender	Male	56	27 (48%)	10 (18%)	19 (34%)	0.13
	Female	14	11 (79%)	1 (7%)	2 (14%)	
Histological grade	G1–G2	37	15 (41%)	10 (27%)	12 (32%)	0.047
	G3	19	11 (58%)	0	8 (42%)	
	unknown	14	12 (86%)	1 (7%)	1 (7%)	
Histology	Adenocarcinoma	15	11 (73%)	0	4 (27%)	0.007
	Squamous cell carcinoma	44	18 (41%)	10 (23%)	16 (36%)	
	Small cell lung cancer	8	8 (100%)	0	0	
	others	3	1 (33%)	1 (33%)	1 (33%)	

These data together suggest that IGF2BP3 is highly expressed in lung cancer tissues at both mRNA and protein levels, especially in squamous cell carcinoma and adenocarcinoma of lung cancers.

### Overexpression of IGF2BP3 promotes lung cancer cell growth *in vitro*

**Figure 2:**
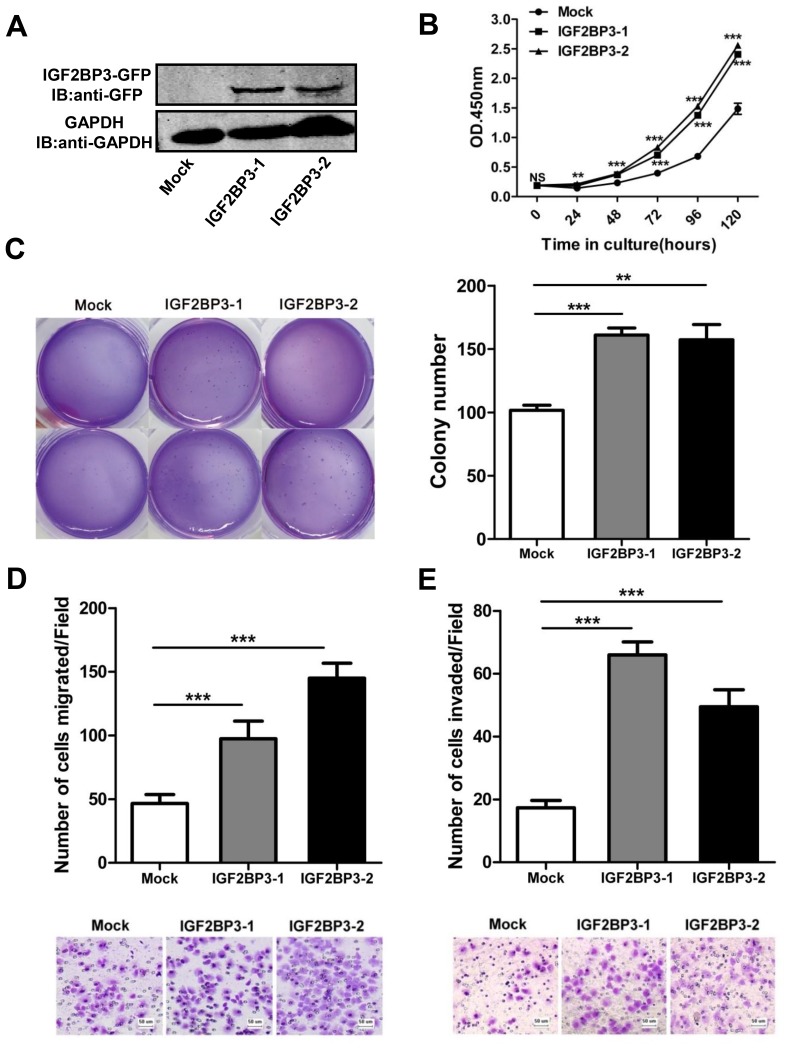
Enhanced cell growth, colony formation, migration and invasion with overexpression of IGF2BP3 pEGFP-N1-IGF2BP3 or pEGFP-N1 were transfected into H460 cells and then the cells were selected with G418 for stably expression cell lines. Single cloned cells named IGF2BP3-1, IGF2BP3-2 and Mock were isolated by limiting dilution assay. (**A**) IGF2BP3 protein expression of single cloned cells was verified by Western blotting with anti-GFP Abs. (**B**) Cell proliferation was analyzed using Cell Counting Kit-8. (**C**) Colony formation was performed in soft agar. (**D** and **E**) Cell migration and invasion assays were performed using Chemotaxis chambers without or with coated Matrigel. The degree of migration and invasion was expressed as the average number of cells in six 20 × fields. Each assay was repeated at least 3 times. Data from one representative experiment are presented as mean ± SD. NS, no significance; **P < 0.01; ***P < 0.001.

The bioinformatics analysis by cBioPortal and GO database showed that the expression of IGF2BP3 in cancer tissues was highly correlated with 395 genes, which mainly participated in biological processes of cell cycle and cell proliferation (Supplementary Figure 3). These data indicated that the increased IGF2BP3 in cancer cells was associated with enhanced proliferation and tumor progression. To elucidate whether IGF2BP3 involved in these processes, we monitored changes in cell behavior following upregulation of its expression in lung cancer cell lines. IGF2BP3 was stably overexpressed in H460 cells (Figure 2A), which had a relatively low level of endogenous IGF2BP3 expression. Two representative clones were used for the following studies. The growth curves of H460 cells with mock or IGF2BP3 overexpression were monitored by CCK-8 assays. As shown in Figure 2B, the growth curves for IGF2BP3 overexpressing cells were significantly higher than those for control cells. Colony formation assay in soft agar showed the similar results (Figure 2C). Besides, we also overexpressed IGF2BP3 in H460 cells by lentiviral transduction (Supplementary Figure 4A), as shown in Supplementary Figure 4B and 4C, lentiviral-mediated overexpression of IGF2BP3 promotes the cell proliferation of H460 cells as well. These data together suggest that overexpression of IGF2BP3 in lung cancer cells facilitates cell proliferation.

### Overexpression of IGF2BP3 promotes lung cancer cell migration and invasion

Tumor migration and invasion are also important features of tumorigenesis. Here we further explored the impact of IGF2BP3 on lung cancer migration and invasion. Transwell assays showed that overexpression of IGF2BP3 in H460 cells led to a significant enhanced lung cancer cell migration compared to mock control (Figure 2D and Supplementary Figure 4D). When the transwell membrane was coated with matrigel, IGF2BP3 overexpressing lung cancer cells showed increased ability to migrate through the membrane (Figure 2E and Supplementary Figure 4D). These data together suggest that overexpression of IGF2BP3 contributes to lung cancer cell migration and invasion.

### Overexpression of IGF2BP3 promotes lung cancer cell growth *in vivo*

**Figure 3:**
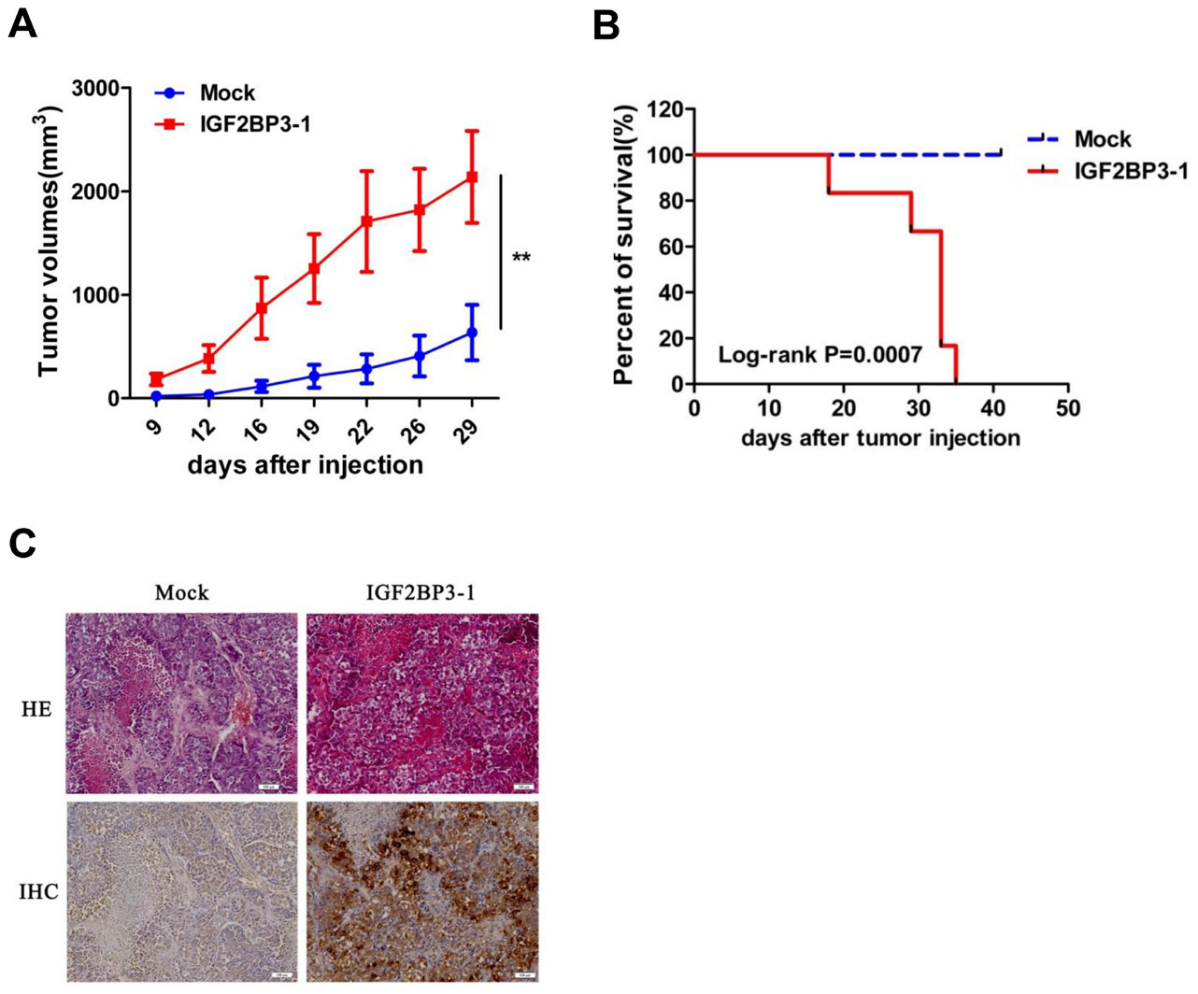
Increased tumorigenicity of H460 cells *in vivo* with overexpression of IGF2BP3 BALB/c nude mice received a subcutaneous injection of 2 × 10^6^ H460 cells stably transfected with IGF2BP3 (IGF2BP3-1) or Mock, and were sacrificed at day 41 after tumor inoculation (*n* = 6 for each group). (**A**) Growth curves showing changes in tumor volume. (**B**) Kaplan-Meier survival curves in Mock and IGF2BP3 transfected mice. (**C**) HE staining and Immunohistochemical staining of IGF2BP3 expression by anti-IGF2BP3 antibody in tumor tissues. Magnification, ×100. **P < 0.01.

Next, *in vivo* subcutaneous tumor formative assay was adopted to examine the tumorigenesis of H460 cells with IGF2BP3 expression in nude mice. Compared to control cells, the injection of IGF2BP3 overexpressing lung cancer cells led to a significantly increase in tumor volume (Figure 3A). The survival rate was also monitored. At day 35, all mice in IGF2BP3 overexpressing group died. In sharp contrast, all control mice were alive at this time course (Figure 3B). The enhanced expression of IGF2BP3 protein in resected tumors from nude mice was verified by immunochemical staining against IGF2BP3 (Figure 3C).

### Overexpression of IGF2BP3 facilitates lung cancer cell metastasis *in vivo*

**Figure 4:**
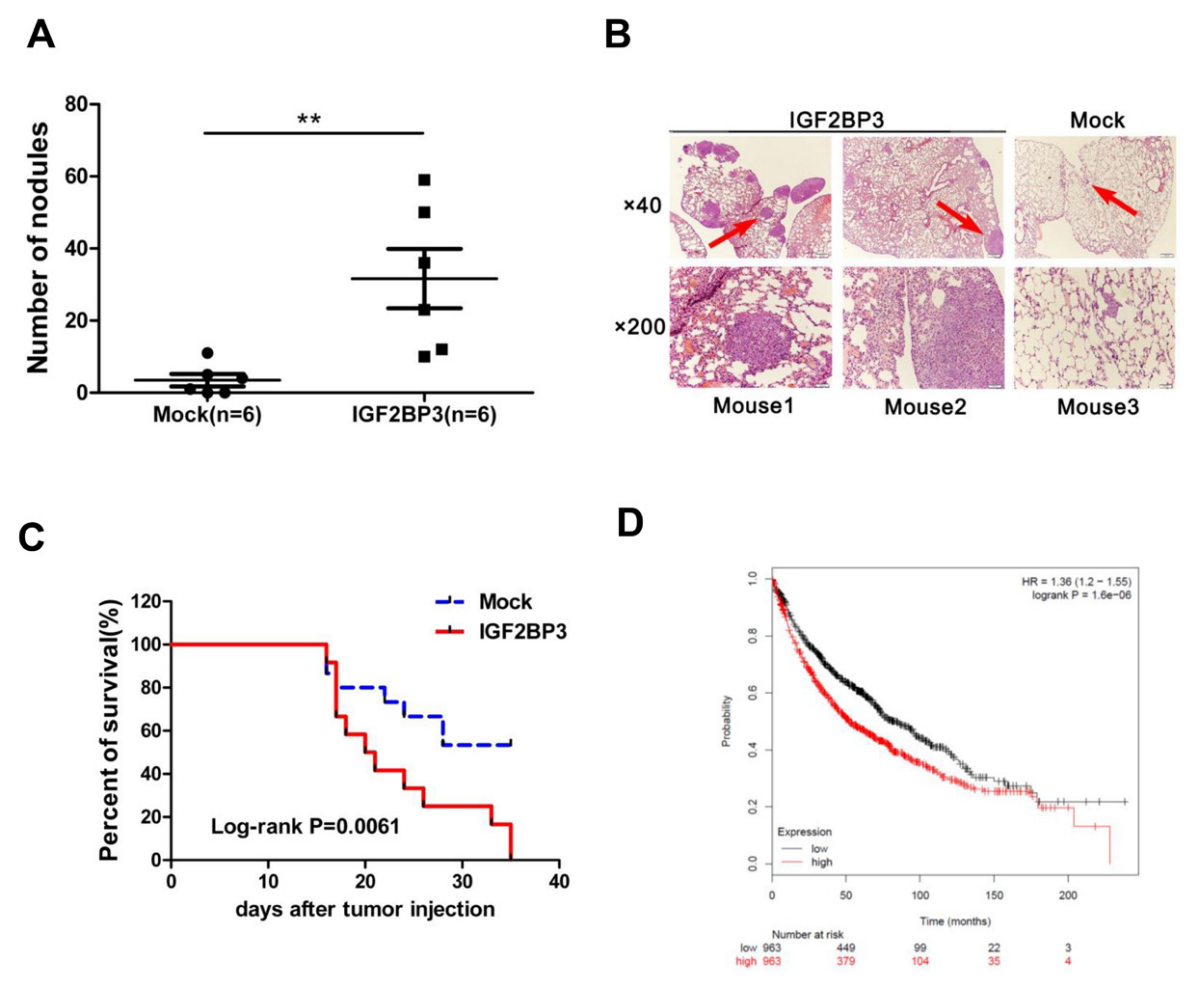
Overexpression of IGF2BP3 promotes lung cancer metastasis and limits survival time H460 cells (1.8 × 10^6^) infected with Mock or IGF2BP3 lentiviruses were injected into the tail vein of BALB/c nude mice. (**A**) Metastasis nodules in the Mock (*n* = 6) and IGF2BP3 (*n* = 6)-expressing H460 tumors were quantified. The horizontal and error bars show the average number and standard deviation. (**B**) H and E staining of lung tissues. Three representative fields are shown. Magnification, ×40, ×200. (**C**) Kaplan-Meier survival curves in mice with Mock (*n* = 15) and IGF2BP3 (*n* = 12) -expressing H460 injection. (**D**) Kaplan-Meier survival analysis for the relationship between survival time and IGF2BP3 expression in lung cancer using the online tool Kaplan-Meier Plotter (http://kmplot.com/analysis/), which is capable to assess the effect of 54675 genes on survival using 10641 cancer samples. The red and black numbers at the bottom represent the total number of cancer patients who have not yet died at the indicated time corresponding to the horizontal axis. **P < 0.01.

In an established model of metastatic lung cancer, H460 cells with IGF2BP3 overexpression were injected into the tail vein of nude mice. Animals were sacrificed at day 21 and the lungs were resected and photographed. The number of tumor nodules in lungs was significantly enhanced in IGF2BP3 overexpressing group compared with control group (Figure 4A and 4B). In agreement with the subcutaneous lung cancer model, IGF2BP3 overexpressing group in the metastatic lung cancer model also showed reduced survival rate (Figure 4C). To support this, we also analyzed the survival rate of lung cancer patients by an online tool “Kaplan-Meier Plotter” analysis (http://kmplot.com/analysis/) [34]. As shown in Figure 4D, lung cancer patients with high expression of IGF2BP3 had shorter survival rate compared to those with low expression of IGF2BP3.Thus, both *in vitro* and *in vivo* assays suggest that overexpression of IGF2BP3 promotes proliferation, metastasis and tumorigenicity of lung cancer cells.

### Knockdown of IGF2BP3 expression inhibits cell growth, migration and invasion

**Figure 5:**
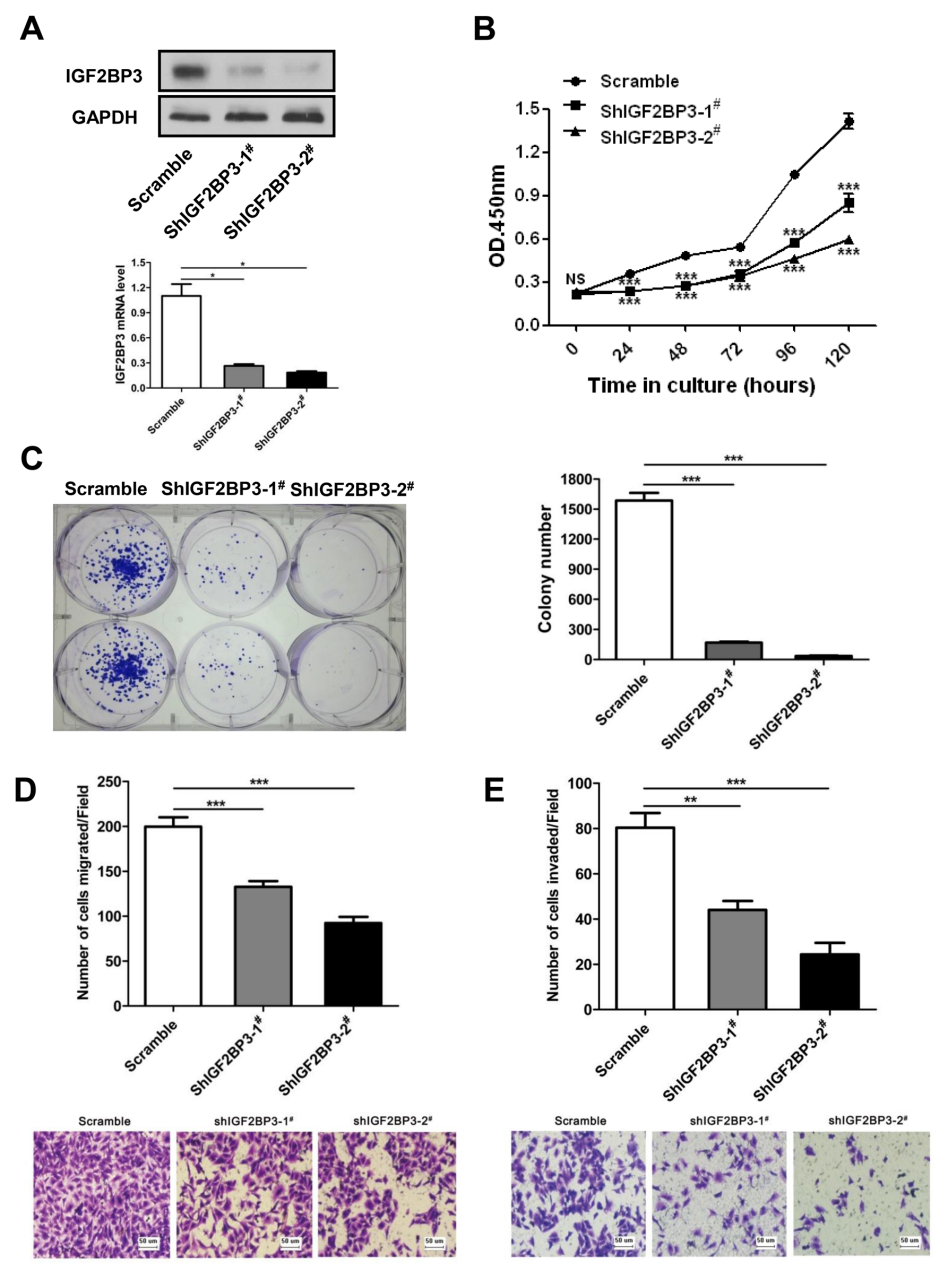
Reduced cell growth, colony formation, migration and invasion upon silencing of IGF2BP3 expression A549 cells were infected with lentiviruses containing scramble or IGF2BP3 shRNA to knockdown endogenous IGF2BP3 expression. (**A**) Knockdown efficiency was monitored in scramble and shIGF2BP3-1^#^ & 2^#^ infected A549 cells. (**B**) Cell proliferation was detected by Cell Counting Kit-8. (**C**) Colony formation was performed in monolayer culture. Cell migration (**D**) and invasion assays (**E**) were performed using Chemotaxis chambers with or without coated Matrigel. The degree of migration and invasion was expressed as the average number of cells in six 20× fields. Each assay was repeated at least 3 times. Data from one representative experiment are presented as mean ± SD. NS, no significance; *P < 0.05; **P < 0.01; ***P < 0.001.

To knockdown endogenous IGF2BP3 expression in lung cancer cells, short hairpin RNA (shRNA) against IGF2BP3 was designed and inserted into the lentiviral shRNA plasmid. The knockdown efficiency was verified by Western blot and Real-time PCR. As shown in Figure 5A, IGF2BP3 protein and mRNA levels were significantly reduced in IGF2BP3 knockdown A549 cells. Then CCK-8 assay was used to assess the proliferation potential of A549 cells with IGF2BP3 knockdown. Compared to control cells, the growth rates for IGF2BP3 knockdown cells were significantly reduced (Figure 5B). Additionally, the colony formation assay showed that the number of surviving colonies of IGF2BP3 knockdown cells was dramatically reduced (Figure 5C). In HCT116 cells, knockdown of IGF2BP3 expression had the similar results on cell proliferation and colony formation (Supplementary Figure 5.). The migration and invasion potential of IGF2BP3 knockdown cells were also evaluated. As anticipated, knockdown of IGF2BP3 expression resulted in decreased cell migration and invasion (Figure 5D and 5E). These data together indicate that knockdown of IGF2BP3 in lung cancer cells suppresses cell proliferation, migration and invasion.

### USP10 is a binding partner of IGF2BP3

**Figure 6:**
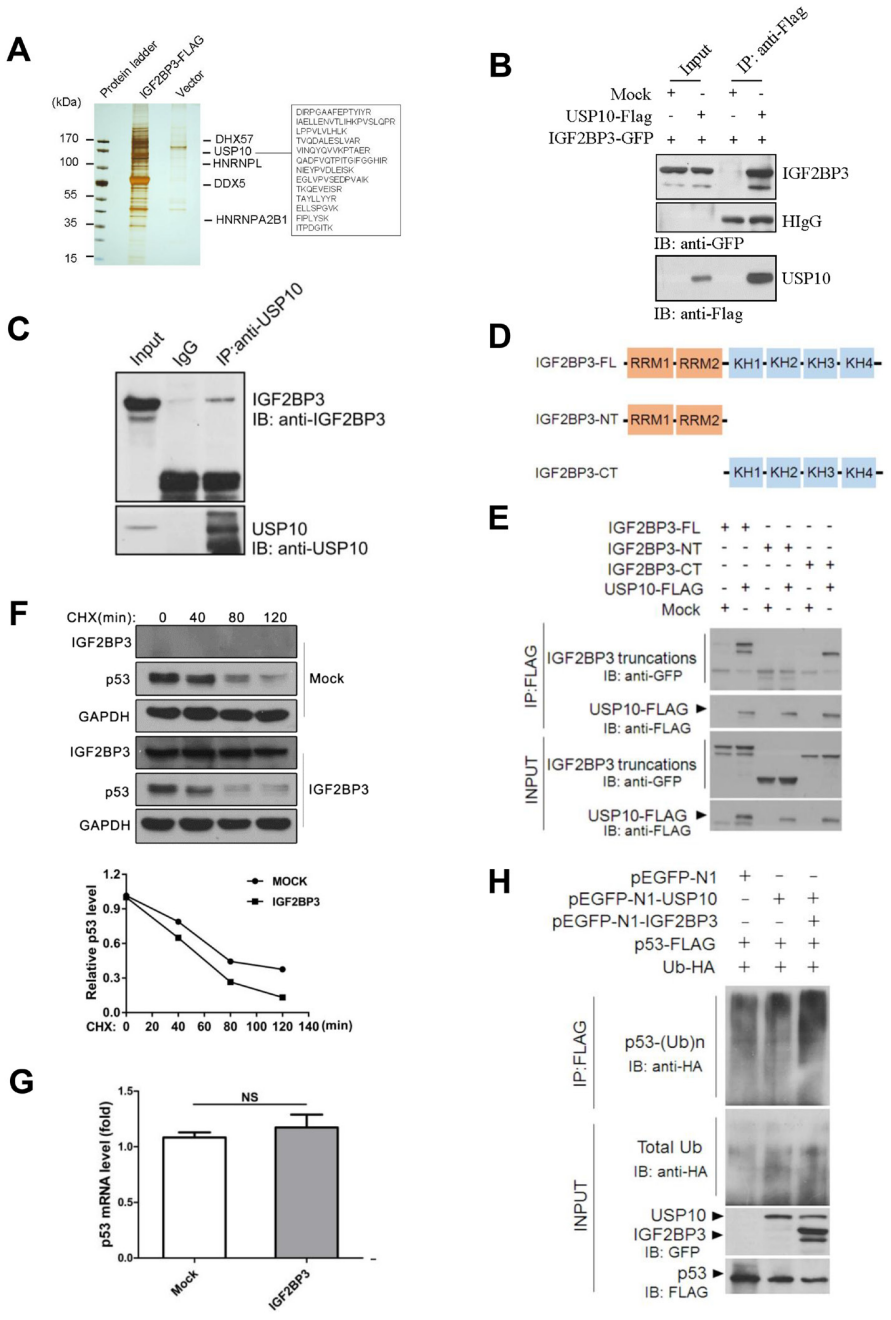
IGF2BP3 physically interacts with USP10 and attenuates USP10 mediated deubiquitination of p53 (**A**) IGF2BP3-FLAG pull-down products from HEK293T cells were separated by SDS-PAGE and visualized by silver staining. IGF2BP3-interacting proteins were identified by mass spectrometry. (**B**) USP10-Flag plasmid was transiently transfected with IGF2BP3-GFP into HEK293T cells. Their interactions were examined by co-IP with anti-Flag Abs and by western blotting with anti-GFP Abs. (**C**) Endogenous interaction of IGF2BP3 and USP10 in HEK293T cells. Cell lysates were immunoprecipitated with anti-USP10 specific Abs using normal rabbit IgG as a control and the presence of IGF2BP3 in the precipitate was determined with anti-IGF2BP3 Abs. (**D**) Domain structures of IGF2BP3 and its truncated mutants. (**E**) USP10-FLAG plasmid was cotransfected with IGF2BP3 or with each of its truncated mutants. Their interactions were determined as described in (B). (**F**) The H460 cells infected with Mock and IGF2BP3 lentiviruses were treated with cycloheximide (CHX) for indicated timepoint. The protein levels in the treated cells were determined by western blotting using anti-Flag(IGF2BP3) and anti-p53. GAPDH was used as a loading control. (**G**) p53 mRNA level in Mock and IGF2BP3 infected H460 cells. Experiments were repeated in triplicate. Data from one representative experiment are presented as mean ± SEM. NS, no significance. (**H**) Mock, IGF2BP3, USP10, p53 and ubiquitin expressing plasmids were cotransfected with indicated constructs into HEK293T cells. p53 ubiquitination in transiently transfected cells was analyzed by immunoprecipitating the lysate with anti-Flag Abs and by western blotting with anti-HA antibody.

To investigate the molecular mechanism by which IGF2BP3 promoted lung tumorigenesis, we performed immunoprecipitation assay followed by mass spectrometry (MS) to identify IGF2BP3-assoicated proteins. As shown in Figure 6A, we obtained several IGF2BP3-associated proteins, including DHX57, HNRNPL, HNRNPA2B1. HNRNPL is known as an IGF2BP3 interacting protein, which suggested that our MS analysis was successful. The IGF2BP3 interaction network was drawn from reported and our MS data (Supplementary Figure 6). Interestingly, a deubiquitinase USP10 was also identified as a binding partner of IGF2BP3. Thirteen unique peptides belonging to USP10 were identified (Figure 6A). Further co-immunoprecipitation assay confirmed the interaction between IGF2BP3 and USP10 (Figure 6B). Moreover, the physiological interaction between the endogenous IGF2BP3 and USP10 was verified in HEK293T cells (Figure 6C). Then, to answer which domain of IGF2BP3 is involved in the interaction between IGF2BP3 and USP10, we constructed truncated mutants of IGF2BP3 (Figure 6D). Co-IP and western blotting analysis revealed that the C-terminal KH domains of IGF2BP3 are required for their interaction with USP10, as deletion of these regions completely abolished their interaction with USP10 (Figure 6E). These data together indicate that USP10 is a binding partner of IGF2BP3 by its C-terminal KH domains.

### IGF2BP3 reduces p53 stability by attenuating the deubiquitination activity of USP10 on p53

Previously, USP10 was reported to be an essential component to control p53 ubiquitination and degradation [35]. Here we asked whether IGF2BP3 regulated the p53 expression level by USP10. In IGF2BP3 overexpressing cells, the half-life of the p53 protein was decreased (Figure 6F). In contrast, the mRNA expression levels were not changed in both groups (Figure 6G). Further experiments were performed to monitor the ubiquitination level of the p53 protein. And the results showed that compared with USP10 alone group, IGF2BP3 coexpression promoted the ubiquitination level of p53 (Figure 6H). These data together suggest that IGF2BP3 reduces p53 level by attenuating the deubiquitinating effect of USP10 on p53.

### IGF2BP3 promotes tumorigenesis by attenuating p53 stability

**Figure 7:**
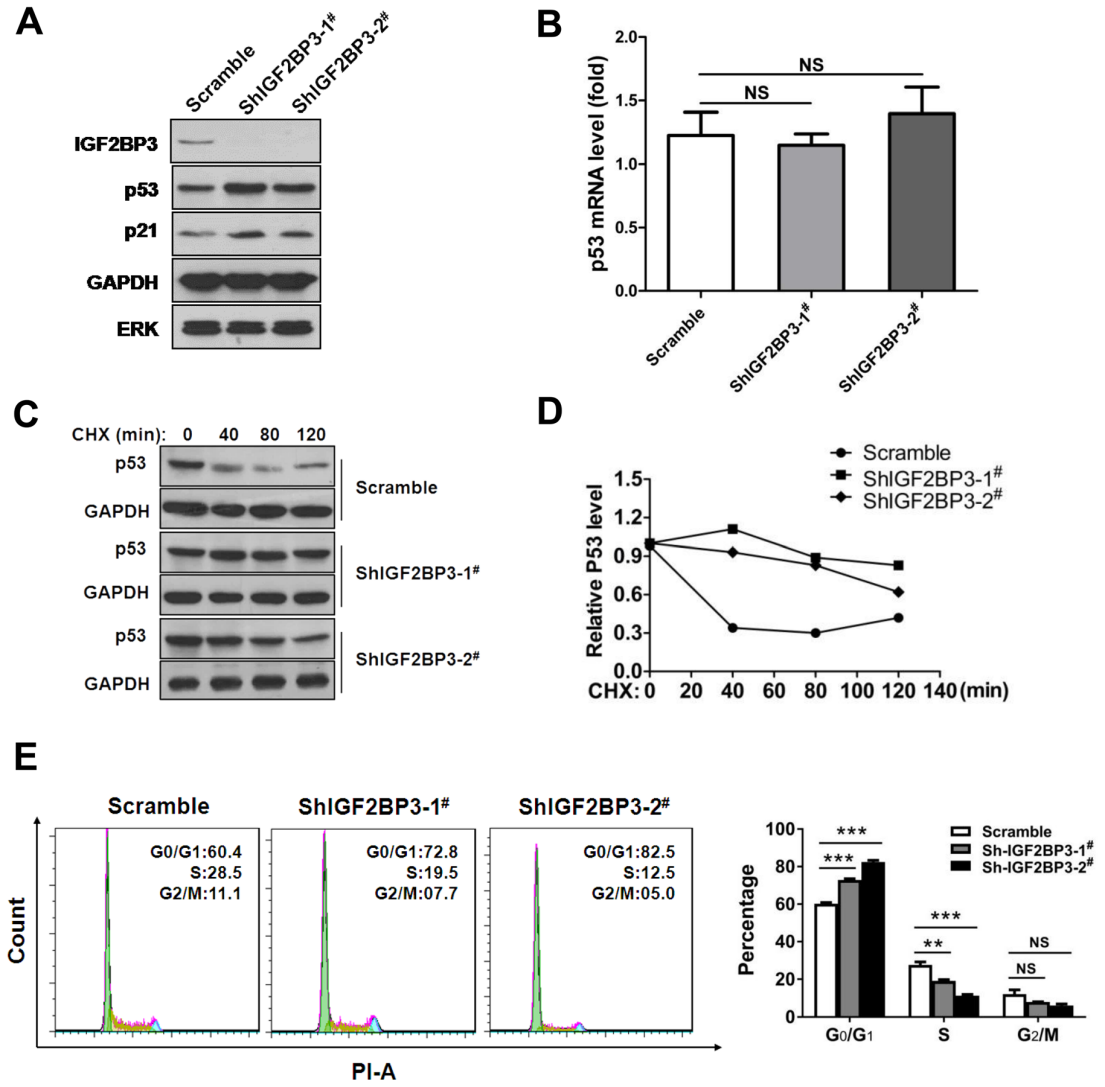
Knockdown of IGF2BP3 increases the stability of p53 protein (**A**) Knockdown of IGF2BP3 in A549 upregulated the protein level of p53. (**B**) p53 mRNA level in scramble or IGF2BP3 knockdown A549 cells. Experiments were repeated in triplicate. Data from one representative experiment are presented as mean ± SEM. NS, no significance. (**C** and **D**) The half-life of p53 was increased in IGF2BP3 knockdown cells. The scramble or IGF2BP3 knockdown A549 cells were treated with cycloheximide (CHX), then the protein level of p53 was monitored. The band intensities of p53 proteins were quantified, and their relative levels are shown in (D). (**E**) Knockdown of IGF2BP3 in A549 cells resulted in G0/G1 arrest. NS, no significance; **P < 0.01; ***P < 0.001.

To determine the impact of IGF2BP3 on p53 level, we also analyzed the p53 expression in lung cancer cells with IGF2BP3 knockdown. In IGF2BP3 silencing A549 cells, we observed a marked increase of p53 protein level, concurrent with p21 upregulation (Figure 7A). Notably, the mRNA expression of p53 was not changed in IGF2BP3 knockdown cells compared to control cells (Figure 7B). Consistently, the half-life of the p53 protein in IGF2BP3 knockdown A549 cells was longer than the control cells (Figure 7C and 7D). Next, we explored whether the change of p53 protein level contributed to the tumorigenesis of lung cancer cells. Concurrent with the increase of p53 expression, IGF2BP3 knockdown cells exhibited G0/G1 cell cycle arrest (Figure 7E).

To further determine whether the function of IGF2BP3 is dependent on p53, we used doxorubicin and etoposide, as DNA damage agents which can induce p53 activation [36, 37], to treat A549 (p53 positive) and H1299 (p53 negative) lung cancer cell lines in the presence of IGF2BP3 or not. As shown in Supplementary Figure 7A, the protective effects of IGF2BP3 on cell viability against DNA damage agent treatment were detected in A549 cells with WT p53, but not in H1299 cells which is p53 negative. Consistently, overexpression of IGF2BP3 inhibited p53 activity and suppressed the expression of its transcriptional target p21 in A549 cells treated with DNA damage agents (Supplementary Figure 7B). These data together demonstrate that the tumor-promoting effect of IGF2BP3 is dependent on p53 status.

## DISCUSSION

Previously, our group demonstrated that IGF2BP3 is a tumor antigen of diagnostic value for lung cancer [32]. In the present study, we systematically explored the participation of IGF2BP3 in lung cancer development. Experimentally, we indeed observed that IGF2BP3 was highly expressed in lung cancer tissues both at mRNA and protein level. By tissue microarray, we found that consistent with a previous study [29], IGF2BP3 is more likely to be expressed in squamous cell carcinoma and adenocarcinoma. Notably, our data demonstrated that IGF2BP3 is prone to be expressed in high grade of lung cancer tissues. This notion was supported by a previous single immunohistochemical study, in which it was reported that IGF2BP3 may be a potential diagnostic marker for high grade of lung cancers [29]. Therefore, both autoantibodies against IGF2BP3 in lung cancer patients and the high expression of IGF2BP3 in lung cancer tissues may be of diagnostic value for lung cancer. Previously, IGF2BP3 has been reported to be a diagnostic tool for bile duct carcinoma and glioblastoma [38, 39]. In future, whether IGF2BP3 is a useful diagnostic marker to predict malignant status or distinguish malignant tumors from benign tumors in diverse tumors will be intriguing.

The dysregulation of IGF2BP3 expression in cancer tissue suggests a potential role for this molecule in tumorigenesis. Consistent with this concept, overexpression of IGF2BP3 enhanced tumor cell growth, migration and invasion, whereas knockdown of IGF2BP3 expression displayed opposite effects. Furthermore, overexpression of IGF2BP3 promoted the tumorigenesis of lung cancer cells and lung metastasis *in vivo*, leading to decreased survival rate. Kaplan-Meier database suggests that lung cancer patients with high IGF2BP3 has relatively short survival time. These data suggest that IGF2BP3 expression may associate with poor prognosis in lung cancer. IGF2BP3 has been reported to serve as an independent prognostic marker in a variety of cancers including ovarian carcinoma, astrocytoma, colorectal cancer, oral carcinoma, endometrial carcinoma and pancreatic ductal adenocarcinoma [10, 12, 38, 40–42]. Our data extend the knowledge that IGF2BP3 may also be a prognostic marker in lung cancer. By knocking down the expression of IGF2BP3 in A549 and HCT116 cells, the proliferation, migration and invasion potential of these cells were dramatically inhibited. Thus, control of the expression of IGF2BP3 may be a potential therapeutic target for cancer control.

Previously, the tumor-promoting activity of IGF2BP3 was focused on its mRNA activity. However, little is known about the RNA-binding independent mechanism of IGF2BP3 in tumorigenesis. In our study, we performed immunoprecipitation assay followed by mass spectrometry. Several candidate binding partners were identified. Notably, the deubiquitinase USP10 was identified as a candidate IGF2BP3 binding partner. Subsequent co-immunoprecipitation assays confirmed the physiological interaction between IGF2BP3 and USP10. Furthermore, we revealed that the KH domains of IGF2BP3 are required for their interaction with USP10. The KH domain is known to be responsible for the recognition and nucleic acid binding [43]. However, the involvement of KH domains in protein-protein interaction was also reported [44, 45]. Our data was another example that KH domains mediate protein-protein interaction. Deubiquitinases are important regulators of protein stability by removing the ubiquitinated chains from the substrates. Therefore, it raised the question whether IGF2BP3 may participate in regulating protein stability via interacting with USP10. As a deubiquitinase, USP10 has different substrates, such as T-bet, TRAF6, NEMO, H2A.Z, *etc*. and has been reported to be involved in multiple biological and pathological processes [46–49]. Of note, USP10 increases p53 stability by counteracting the ubiquitination ability of MDM2 through deubiquitination [35, 50]. Then our following studies focused on whether IGF2BP3 also regulated the protein stability of p53 via USP10. In IGF2BP3 knockdown cells, the protein level of p53 was dramatically increased and the half-life of p53 was longer in these cells. It suggested that IGF2BP3 indeed reduced the protein expression level of p53. The change of p53 level was reflected by the G0/G1 cell cycle arrest in IGF2BP3 knockdown lung cancer cells. Besides, the ubiquitination level of p53 was increased when IGF2BP3 was overexpressed.

The tumor suppressor p53 plays a pivotal role in the regulation of essential functions such as cell cycle, DNA repair, apoptosis, angiogenesis, *etc* [51]. The deregulation of p53 expression and function was associated with multiple cancers. Here, we defined a novel mechanism by which IGF2BP3 reduced the p53 level and promoted the tumorigenesis of lung tissues. This function is independent of its mRNA binding activity. IGF2BP3 and IGF2BP1 share amino acid homology and functional similarity. It will be of interest in the future to test whether IGF2BP1 possesses the similar function on attenuating p53 stability.

The important point we should keep in mind is that IGF2BP3 is a crucial transcriptional modulator which binds a variety of mRNAs and modulates the gene expression. By known validated target mRNAs, IGF2BP3 can exert its activities in tumor initiation, proliferation and invasion. Here, we uncovered an unknown function of IGF2BP3 to be as a posttranslational modulator in lung cancer development. USP10, as a deubiquitinase, has multiple targets [46–49]. Besides p53, it is possible, other substrates of USP10 may also be regulated by IGF2BP3 and take part in the tumorigenesis. We propose that the transcriptional and posttranslational modulation may work together for IGF2BP3 to function as a potential oncogene in cancer development.

In summary, by systematic studies, we revealed a novel mechanism by which IGF2BP3 conferred lung tumorigenesis. These findings together suggest that IGF2BP3 may be a useful therapeutic target for lung cancer in the future.

## MATERIALS AND METHODS

### Cell lines

H460, A549 and H1299 cells were cultured in RPMI 1640 medium supplemented with 10% fetal bovine serum (Life Technologies, Carlsbad, CA, USA). HCT116 and HEK293T cells were maintained in DMEM medium supplemented with 10% fetal bovine serum. All cell lines were cultured at 37°C in a humidified 5% CO_2_ atmosphere.

### Plasmids

pEGFP-N1-IGF2BP3, IGF2BP3 truncated mutants (pEGFP-N1-IGF2BP3-NT, pEGFP-N1-IGF2BP3-CT), pEGFP-N1-USP10, pcDNA3-Flag-IGF2BP3, pcDNA3-Flag-USP10 were constructed by standard molecular biology techniques, all constructs were confirmed by sequencing. Ub-HA and p53-Flag was from Addgene.

### Tissue microarray and immunohistochemistry

A lung cancer tissue microarray was purchased from Chaoying Biotechnology Company (Xi’an, China). Sections were then incubated with anti-IGF2BP3 antibody (Sigma-Aldrich, Saint Louis, MO, USA) (1:200 dilution) as previously described [52]. Staining was evaluated as negative (no staining, staining in < 10% of tumor cells or barely perceptible staining in > 10% of tumor cells), weak positive (weak to moderate staining in 10–30% of tumor cells) and positive (weak to moderate staining in > 30% of tumor cells or strong staining in > 10% of tumor cells). The stained sections were reviewed and scored by a pathologist. Normal human lung tissue was used as the negative control.

### Transfection and selection of stably transfected H460/IGF2BP3 cells

H460 cells (2 × 10^6^) were transfected with 10μg of pEGFP-N1-IGF2BP3 or pEGFP-N1 vector by electroporation (ECM830, BTX, USA) according to the manufacturer’s instructions and diluted to 6-well plates by 1:20 after 2 days of transfection. For stable clones, the cells were cultured in 700 μg/ml G418 (Life Technologies) 24 hours later. After growth for 2 weeks, GFP positive cell clones were screened to 24-well plates in RPMI 1640 with 400 μg/ml G418 and 10% FBS. High IGF2BP3 expression clone was limiting diluted in 96-well plates until a single colony was formed. Single cloned cells named IGF2BP3-1, IGF2BP3-2 and Mock were isolated and grown up. Western blots were used to test for IGF2BP3 level of stably transfected cell lines.

### cDNA microarray and quantitative real-time PCR

A lung cancer cDNA microarray was purchased from SHANGHAI OUTDO BIOTECH CO.,LTD. (Shanghai, China), which contains 15 paired cancer and non-cancerous lung tissues. Total RNA was isolated by TRIZOL reagent (Life Technologies) and then reverse transcribed using the Reverse Transcription System (Promega, Madison, WI, USA) according to the manufacture’s instruction. Quantitative Real-time PCR was carried out on a Bio-Rad Real-Time PCR system. The sequences of the Real-time primers were as follows: IGF2BP3 forward, 5′- CCATAGAAGTTGAGCACTCGGTCC-3′; reverse, 5′- TCTCCACCACTCCATACTGGACTAG -3′. p53 forward, 5′-GGAAATTTGCGTGTGGAGTATTT-3′; reverse, 5′-GTTGTAGTGGATGGTGGTACAG-3′. Primers for GAPDH have been described before [53]. Gene expression was quantified as the level of indicated genes relative to that of GAPDH.

### Western blot analysis

For Western blot analysis, cells were lysed in RIPA buffer (50 mM Tris, pH8.0, 150 mM NaCl, 1% NP-40, 0.5% deoxycholate, 0.1% SDS, 2 mM EDTA, and protease inhibitor cocktail) for 30 min at 4°C and then centrifuged. The equivalent protein extracts were separated on SDS-PAGE and transferred onto nitrocellulose filter. After blocking, blots were probed with indicated antibodies and detected with the Odyssey Imaging System (LICOR Bioscience, Lincoln, NE, USA) or detected by ECL assay. Antibodies used included anti-IGF2BP3 (Sigma-Aldrich), anti-GAPDH (Proteintech Group Inc, Chicago, Illinois, USA), anti-GFP (MBL Inc, Japan), anti-p53 (Proteintech), anti-p21 (Proteintech), anti-ERK (Cell Signaling Technology, Beverly, MA, USA) anti-Flag and anti-HA(Sigma-Aldrich).

### Lentiviral transduction for shRNA and IGF2BP3 overexpression

Scramble shRNA or two shRNA against IGF2BP3 in lentivral vector pLKO.1 was constructed. The targeting sequences against IGF2BP3 were as follows: shIGF2BP3-1^#^: GAAACTTCAGATACGAAATAT; shIGF2BP3-2^#^: AATCGATGTCCACCGTAAAGA; The scramble shRNA targeting sequence is: TGTAATAGTGCGTTCTGGATT.

Then the packaging cell line HEK293T was transfected by pLKO.1 with packaging plasmid psPAX2 and envelope plasmid pMD2.G. The medium was changed 12 h post-transfection. Forty-eight hours later, lentiviral particle solution was harvested and incubated with A549 cells in the presence of 8 μg/ml polybrene (Sigma-Aldrich). Stable knockdown cells were selected by culturing cells in medium containing puromycin.

For lentiviral transduction and selection of IGF2BP3 overexpressing H460 cells. The protocol is similar to the above shRNA selection. The packaging cell line HEK293T were transfected by pCDH-CMV-IGF2BP3-FLAG-EF1-puro with packaging plasmid psPAX2 and envelope plasmid pMD2.G. The stable IGF2BP3 overexpressing cells were also selected by puromycin.

### Cell proliferation assay

H460 or A549 cells were plated into 96-well plates at 3 × 10^3^ cells/well. Cell viability was determined every 24 h by using Cell Counting Kit-8 (CCK-8) (Dojindo Laboratories, Japan) according to the manufacturer’s instructions. Each condition was repeated at least 3 times.

### Colony formation assay

A total of 2000 cells were plated in a 6-well plate and maintained in medium with or without puromycin (Life Techonogies) for 2 weeks. Colonies were fixed with precooled methanol and stained with 0.5% (w/v) crystal violet. Numbers of colonies (≥ 50 cells per colony) were counted under a light microscope. For soft agar colony formation assay, a total of 200 cells were suspended in 1.5 ml RPMI 1640 containing 0.35% agar and 10% fetal bovine serum, then layered on 1.5ml RPMI 1640 containing 0.5% agar and 10% fetal bovine serum in a 6-well plate. Colonies were stained with crystal violet and counted by direct microscopy after 2 to 3 weeks. All the experiments were done in triplicate wells three times.

### Cell migration and invasion assay

For migration assays, H460 cells (5 × 10^4^) in 100 μl serum-free RPMI 1640 were seeded into the upper chamber of a 24-well Transwell chamber (8-μm pore size, Corning Life Sciences, Corning, NY, USA) coated with 10 μg/ml fibronectin (Sigma–Aldrich). 600 μl medium supplemented with 10% FBS in the lower chamber was used as the chemoattractant. After 20 h of incubation at 37°C, cells adherent to the upper surface of the filter were removed using a cotton swab. Cells were fixed with methanol and stained with crystal violet, and the number of cells at the bottom was counted in 6 randomly 20× fields.

Invasion assays were performed using the same procedure as the migration assay, except that the upper surface of the filter was covered with 30 μL Matrigel (0.5 mg/mL; BD Biosciences, San Jose, California, USA). Data are from three experiments done in triplicate.

### Tumor growth in nude mice

Female BALB/C nude mice (6-week old) were purchased from the Academy of military medical sciences (Beijing, china) and raised in a pathogen-free facility at Peking University Health Science Center (Beijing, China). The animal study protocols used were approved by the ethics committee of Peking University Health Science Center and all animals were treated in accordance with the Institutional Animal Care and Use Committee (IACUC). Mock or IGF2BP3-1 cells were resuspended in phosphate-buffered solution and subcutaneously injected into the flanks (2 × 10^6^ cells per mouse) of 8-week-old nude mice. Mouse survival was monitored daily. Tumor volume was measured every 3 days by measuring the length (l) and width (w) and calculated using the following formula:lw (l + w)π/12. Six weeks after injection, mice were sacrificed by cervical dislocation. Tumor tissues were fixed in 4% formalin and evaluated IGF2BP3 expression with anti-IGF2BP3 antibody by immunohistochemistry.

### Tumor metastasis analysis in nude mice

IGF2BP3 overexpressing or control H460 cells were selected as described above. Cells (1.8 × 10^6^) in 0.18 ml PBS were injected into the tail vein of 8-week-old nude mice. Three weeks after injection, mice were sacrificed. The lungs were removed and the number of tumor nodules on the lung surface was counted by Bouin fixative. Tissue sections were also stained with hematoxylin-eosin (H and E) for histologic evaluation. The survival of mice was also monitored and evaluated.

### Co-immunoprecipitation and *in vivo* ubiquitination assay

HEK293T cells were transfected with mock constructs or indicated plasmids. Twenty-four hours post-transfection, the cells were harvested and lysed in RIPA lysis buffer with a cocktail of protease inhibitors (Roche, Basel, Switzerland). Then the cell lysates were immunoprecipitated with IgG or mouse anti-Flag mAb and protein-A Sepharose (GE Healthcare, USA) and the precipitates were analyzed by immunoblot with the indicated antibodies after six times PBST washing. For endogenous immunoprecipitation, cell lysate was prepared from HEK293T cells and immunoprecipitated with anti-USP10 antibody (Cell Signaling Technology, Beverly, MA, USA) and the precipitate was detected with anti-IGF2BP3 antibody.

For *in vivo* ubiquitination assay, HEK293T cells were transfected with indicated plasmids. Then the cells were treated with 20 mM of MG132 (Sigma-Aldrich) for 8 hours before harvest. The cells were lysed in 100 μl Co-IP lysis buffer plus proteinase inhibitor mixture (Roche) ,PMSF and 1% SDS at 4°C for 2 h, then were boiled for 5 mins at 95°C. Then, the lysates were diluted 10 times with RIPA buffer and subjected to immunoprecipitation with mouse anti-Flag mAb and protein-A Sepharose at 4°C. The precipitates were analyzed by immunoblot with anti-HA antibody.

### Mass spectrometry analysis of IGF2BP3 binding proteins

Flag-tagged IGF2BP3 or control constructs were transfected into HEK293T cells. Forty-eight hours post-transfection, the cells were harvested and lysed as above. Then the cell lysates were immunopurified with Flag affinity beads and eluted with Flag peptides. The eluates were resolved in SDS-PAGE. Then the gel was silver- stained. And the excised gel segments were subjected to mass spectrometry analysis. The detailed mass spectrometry method was described previously [54].

### Cell cycle

IGF2BP3 knockdown or control A549 cells (5 × 10^4^) were harvested and fixed in ice cold 70% ethanol for 30 min. After washing with PBS, cell pellets were resuspended in 0.5 ml of PBS containing 10 μg/ml propidium iodide (PI, Sigma-Aldrich) and 500 μg/ml RNase A (Sigma-Aldrich) and incubated at 37°C for 30 min. Samples were then analyzed by FACS Calibur flow cytometry (BD Biosciences) [55].

### Statistical analysis

Statistical analysis was performed on SPSS13.0. Data were expressed as mean ± SD. Differences between two groups were explored by Student’s *t* test. The chi-square test was used to calculate differences in different gender, histological grade and histology of the patient. Significance of IGF2BP3 levels on nude mice and patient survival was analyzed using Kaplan-Meier log-rank test. *P* < 0.05 was considered to be statistically significant.

## SUPPLEMENTARY MATERIALS FIGURES



## Abbreviations

IGF2BP3: Insulin-like growth factor 2 mRNA binding protein 3
USP10: Ubiquitin specific peptidase 10
NSCLC: non-small cell lung cancer
SCLC: small cell lung cancer
ADC: adenocarcinoma
SCC: squamous cell carcinoma
LCLC: large cell lung cancer
PI: propidium iodide
