# Supplementary material for: Insulin-like growth factor 2 mRNA binding protein 3 (IGF2BP3) promotes lung tumorigenesis via attenuating p53 stability
Source: Oncotarget. 2017 Sep 27;8(55):93672–87. doi: 10.18632/oncotarget.21280 (PMC5706827; doi:10.18632/oncotarget.21280)
Supplement: Supplementary file 1 [file oncotarget-08-93672-s001.pdf]

Insulin-like growth factor 2 mRNA binding protein 3 (IGF2BP3) promotes lung tumorigenesis via attenuating p53 stability

SUPPLEMENTARY MATERIALS

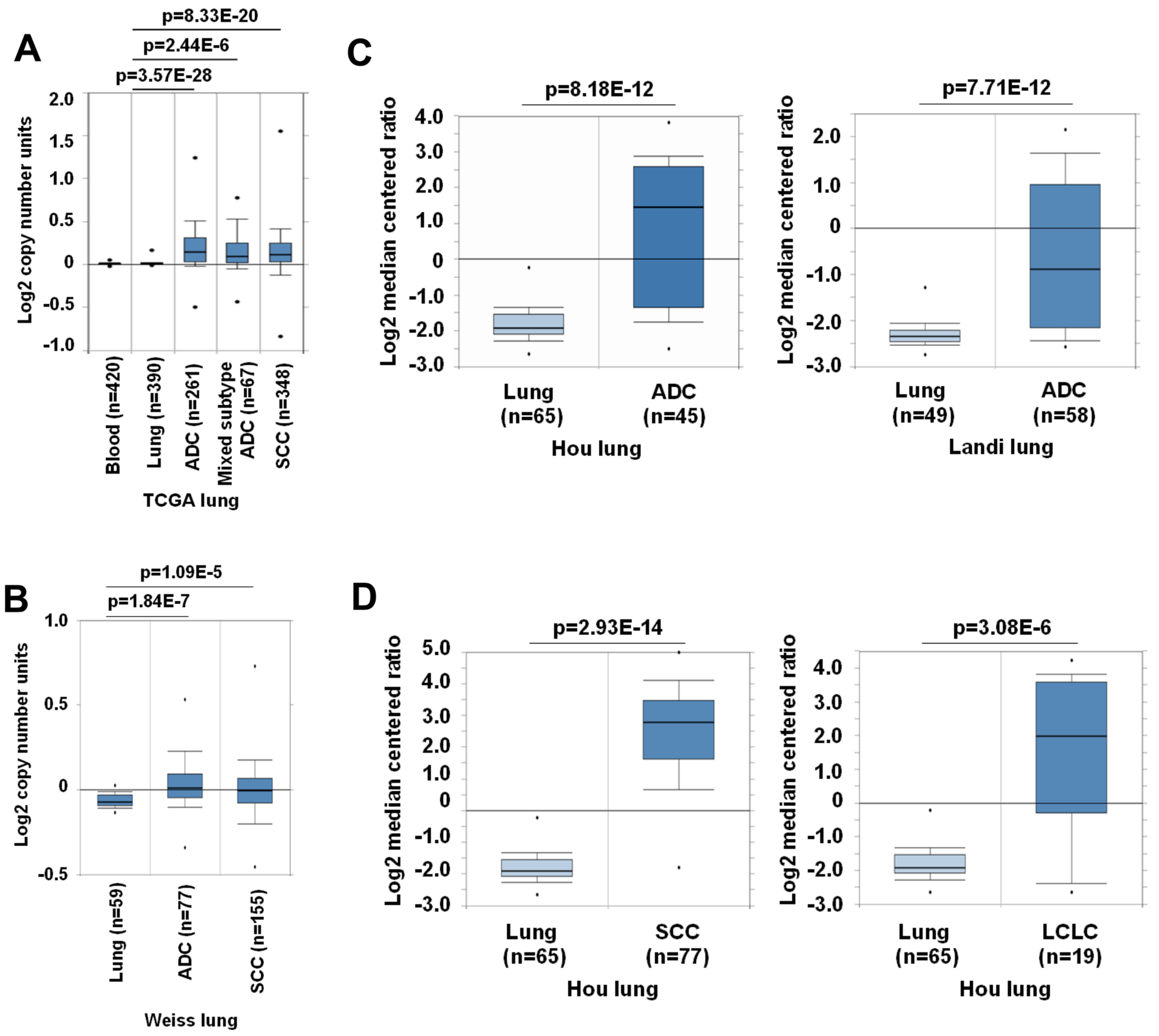

Supplementary Figure 1: Amplification and up-regulation of the IGF2BP3 gene in lung cancer. (**A**) DNA copy numbers of the IGF2BP3 gene were increased in lung carcinoma tissues compared to blood (whole blood genomics DNA) by analyzing TCGA lung database from Oncomine. (**B**) DNA copy numbers of the IGF2BP3 gene were increased in Weiss lung database from Oncomine. (**C**) IGF2BP3 mRNA was upregulated in lung adenocarcinoma by analyzing the Hou and Landai lung database from Oncomine. (**D**) IGF2BP3 mRNA was upregulated in squamous cell lung carcinoma by analyzing the Hou and Landai lung database from Oncomine. ADC, Adenocarcinoma; SCC, Squamous cell carcinoma; LCLC; Large cell carcinoma;

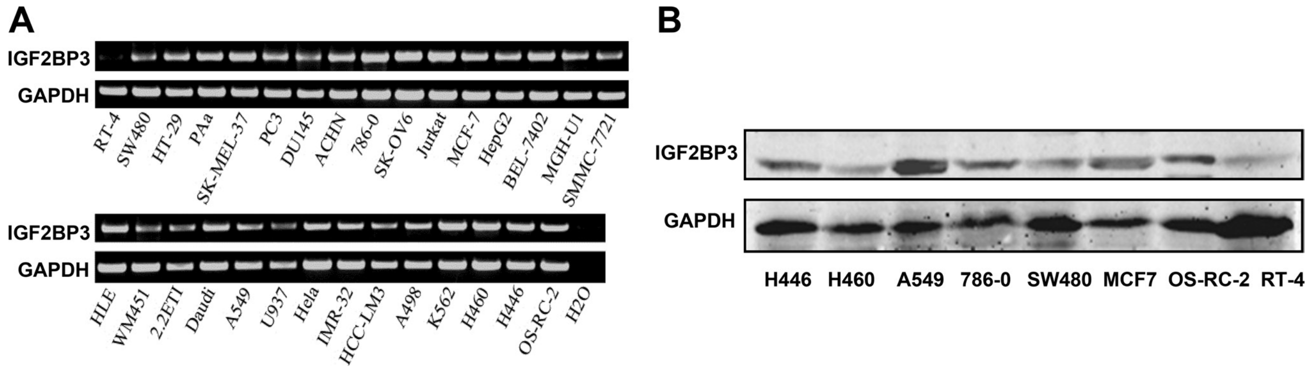

Supplementary Figure 2: IGF2BP3 is ubiquitously increased in cancer cell lines. (**A**) RT-PCR of IGF2BP3 mRNA in cancer cell lines. (**B**) Western blot analysis of IGF2BP3 protein in cancer cell lines.

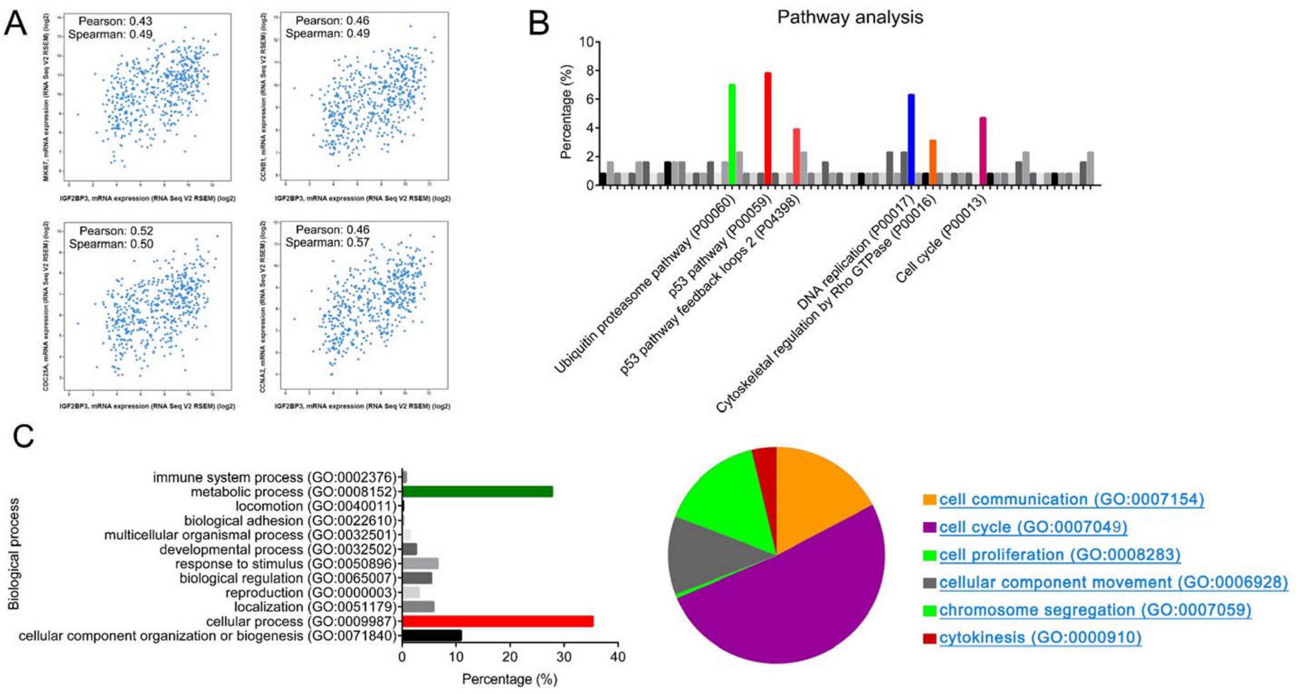

Supplementary Figure 3: Pathway and functional analysis of IGF2BP3-related genes. (**A**) The bioinformatics analysis (www.cbioportal.org) showed that the expression of IGF2BP3 in cancer tissues was highly correlated with 395 genes, including the hallmark of proliferation and cell cycle such as Ki67, CCNA2, CCNB1, et al. (**B** and **C**) Pathway analysis in GO database showed that majority of them were involved in p53 signaling and cell cycle (B) and they mainly participated in biological process of cell cycle and cell proliferation(C).

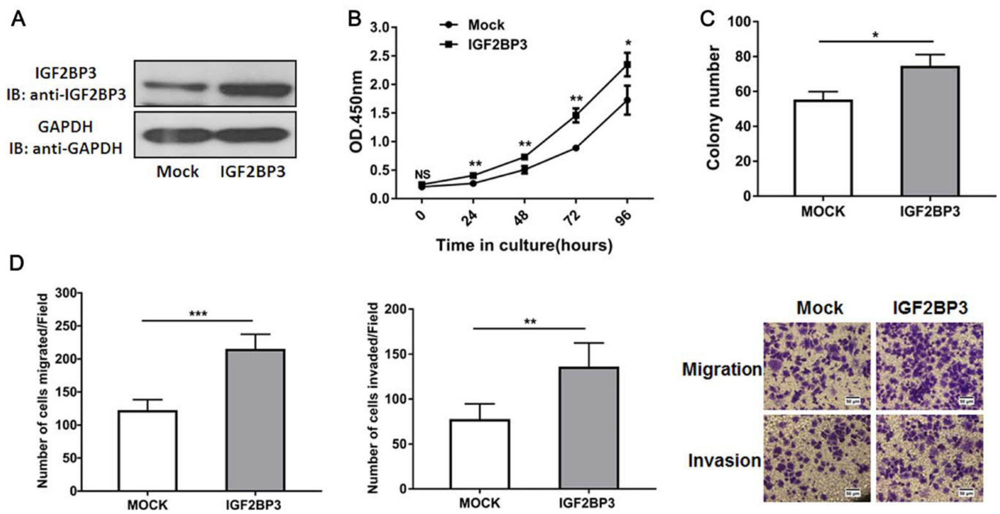

Supplementary Figure 4: Enhanced lung cancer cell growth, colony formation, migration and invasion with overexpression of IGF2BP3 by lentiviral transduction. H460 cells were infected with lentiviruses containing Mock or IGF2BP3 expressing plasmid to enhance IGF2BP3 expression. (**A**) IGF2BP3 protein expression was verified by Western blotting with anti-IGF2BP3 Abs. (**B**) Cell proliferation was analyzed using Cell Counting Kit-8. (**C**) Colony formation was performed in soft agar. (**D**) Cell invasion and migration assays were performed using Chemotaxis chambers with or without coated Matrigel. The degree of migration and invasion was expressed as the average number of cells in six 20 × fields. Each assay was repeated at least 3 times. Data from one representative experiment are presented as mean ± SD. NS, no significance; **P* < 0.05; ***P* < 0.01; ****P* < 0.001.

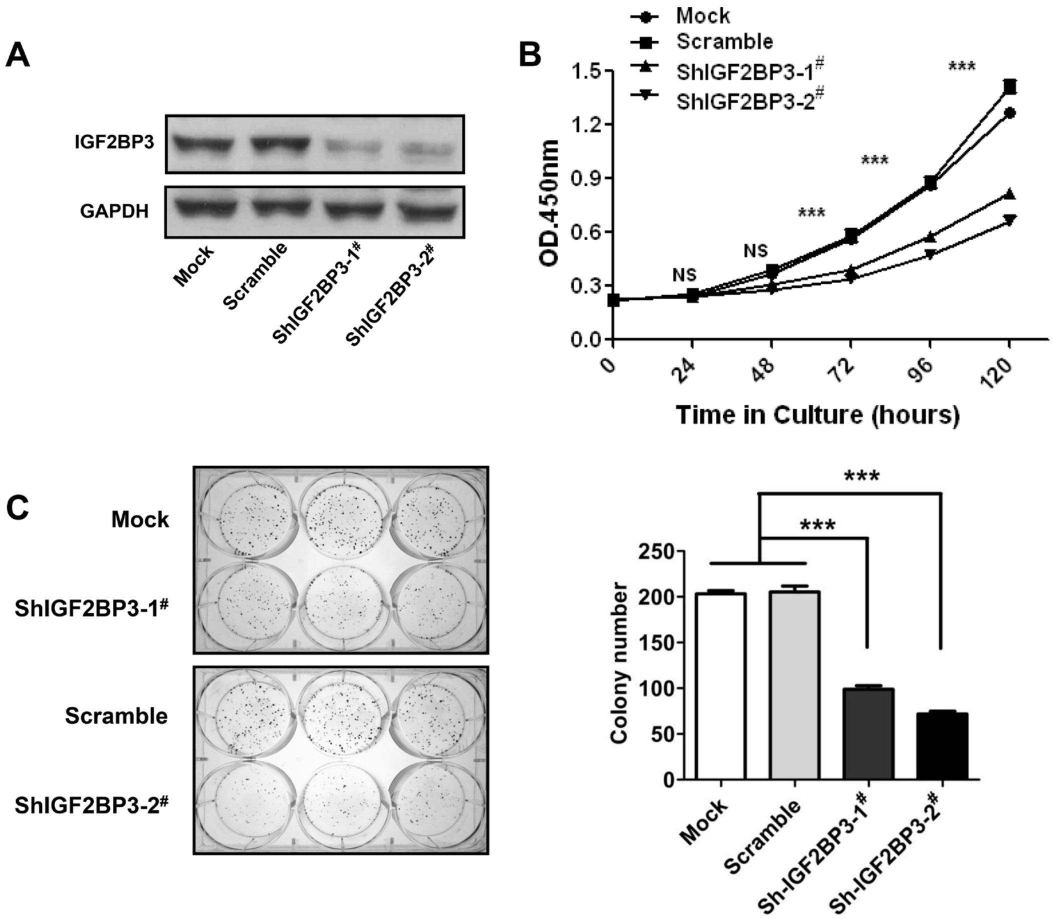

Supplementary Figure 5: Reduced cell growth in IGF2BP3 silencing HCT116 cells. HCT116 cells were infected with lentiviruses containing scramble or IGF2BP3 shRNA to knockdown endogenous IGF2BP3 expression. (**A**) Knockdown efficiency was monitored in scramble and shIGF2BP3-1# & 2# infected HCT116 cells. (**B**) Cell proliferation was detected by Cell Counting Kit-8. (**C**) Colony formation was performed in monolayer culture. NS, no significance; ****P* < 0.001.

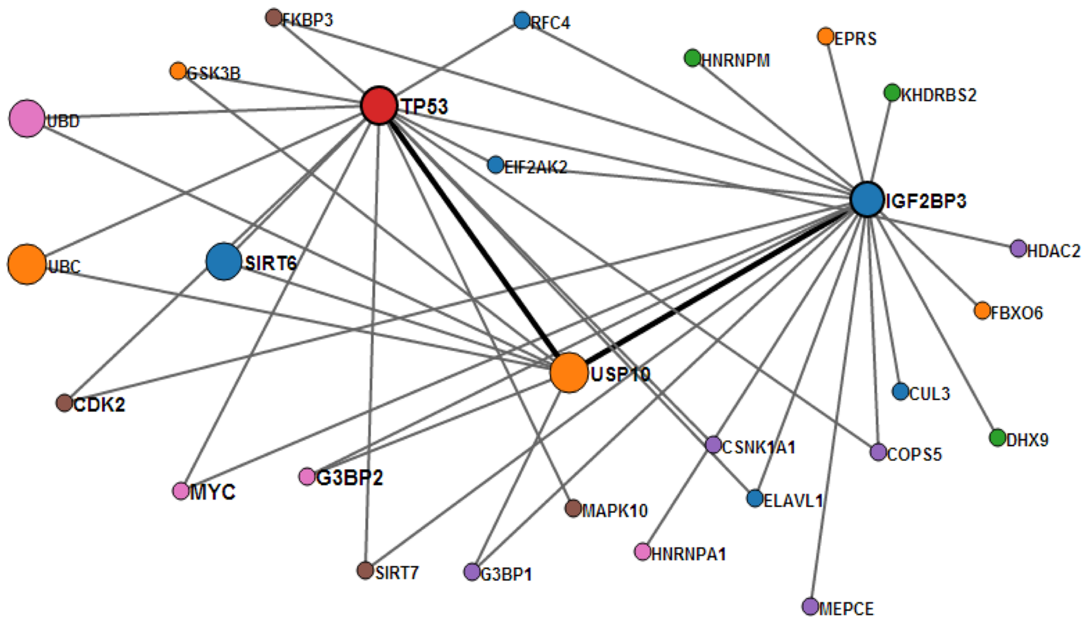

Supplementary Figure 6: Network of IGF2BP3 associated proteins (known and our data).

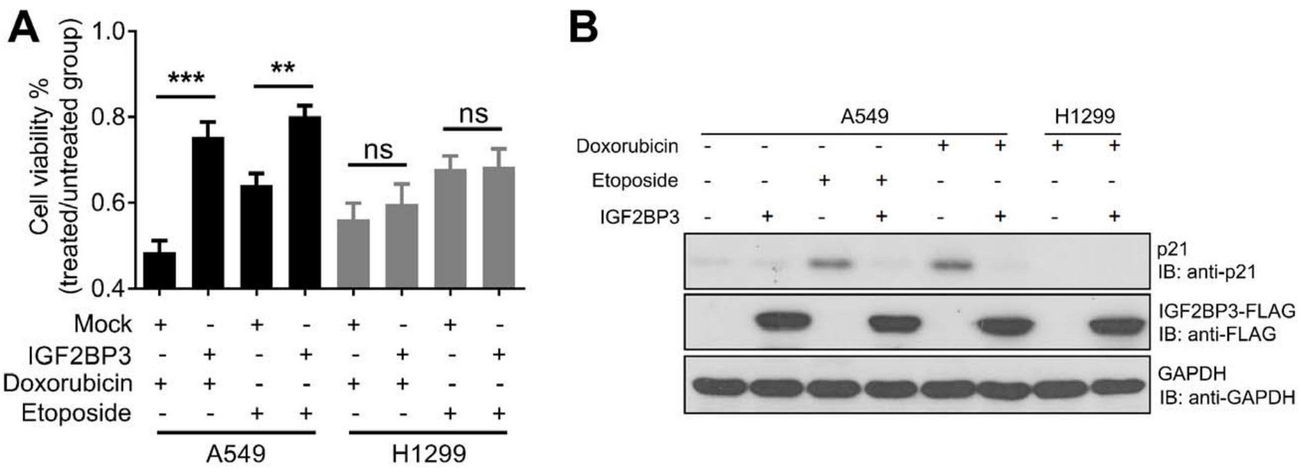

Supplementary Figure 7: IGF2BP3 enhances chemoresistance to doxorubicin and etoposide in a p53 dependent manner. A549 (p53 positive) and H1299 cells (p53 negative) were transfected with Mock or IGF2BP3 plasmid. 24 h later, they were treated with doxorubicin (0.4 μg/ml) or etoposide (50 μM) for 24 h. (**A**) The chemosensitivity to doxorubicin and etoposide was measured by CCK8. NS, no significance; $^{**}P < 0.01$; $^{***}P < 0.001$. (**B**) protein expression was verified by Western blotting.
